# Associations of Human Papillomavirus (HPV) genotypes with high-grade cervical neoplasia (CIN2+) in a cohort of women living with HIV in Burkina Faso and South Africa

**DOI:** 10.1371/journal.pone.0174117

**Published:** 2017-03-23

**Authors:** Helen A. Kelly, Jean Ngou, Admire Chikandiwa, Bernard Sawadogo, Clare Gilham, Tanvier Omar, Olga Lompo, Sylviane Doutre, Nicolas Meda, Helen A. Weiss, Sinead Delany-Moretlwe, Michel Segondy, Philippe Mayaud, for the HARP Study Group

**Affiliations:** 1 Clinical Research Department, London School of Hygiene and Tropical Medicine, London, United Kingdom; 2 INSERM U1058 and University Hospital (CHRU), Montpellier, France; 3 Wits Reproductive Health and HIV Institute, University of the Witwatersrand, Johannesburg, South Africa; 4 Centre de Recherches Internationales en Santé, University of Ouagadougou, Ouagadougou, Burkina Faso; 5 National Health Laboratory Services, Johannesburg, South Africa; 6 Department of Infectious Disease Epidemiology, London School of Hygiene and Tropical Medicine, London, United Kingdom; 7 MRC Tropical Epidemiology Group, London School of Hygiene and Tropical Medicine, London, United Kingdom; Hôpital Bichat-Claude Bernard, FRANCE

## Abstract

**Objective:**

To describe associations of high-risk human papillomavirus (HR-HPV) with high-grade cervical intraepithelial neoplasia (CIN2+) in women living with HIV (WLHIV) in Burkina Faso (BF) and South Africa (SA).

**Methods:**

Prospective cohort of WLHIV attending HIV outpatient clinics and treatment centres. Recruitment was stratified by ART status. Cervical HPV genotyping using INNO-LiPA and histological assessment of 4-quadrant cervical biopsies at enrolment and 16 months later.

**Results:**

Among women with CIN2+ at baseline, the prevalence of any HR-HPV genotypes included in the bi/quadrivalent (HPV16/18) or nonavalent (HPV16/18/31/35/45/52/58) HPV vaccines ranged from 37% to 90%. HPV58 was most strongly associated with CIN2+ (aOR = 5.40, 95%CI: 2.77–10.53). At 16-months follow-up, persistence of any HR-HPV was strongly associated with incident CIN2+ (aOR = 7.90, 95%CI: 3.11–20.07), as was persistence of HPV16/18 (aOR = 5.25, 95%CI: 2.14–12.91) and the additional HR types in the nonavalent vaccine (aOR = 3.23, 95%CI: 1.23–8.54).

**Conclusion:**

HR-HPV persistence is very common among African WLHIV and is linked to incident CIN2+. HPV vaccines could prevent between 37–90% of CIN2+ among African WLHIV.

## Introduction

Women living with HIV (WLHIV) have a higher prevalence of genital high-risk human papillomavirus (HR-HPV) infection than the general population [1], are more likely to be infected with multiple HR types [2, 3] and have greater persistence of infection [4] and risk of cervical intraepithelial neoplasia (CIN) progression [5]. WLHIV have been shown to be more commonly infected with types other than HPV16 or 18 [6] and their high-grade cytological lesions are frequently attributed to types other than HPV16/18 [3].

As WLHIV are living longer due to increased availability of antiretroviral therapy (ART), many retain a high risk of infection with HPV and high risk of progressing to cervical and other genital cancers. Primary prevention of HPV infection through vaccination could reduce the burden of infection and disease and on screening and treatment services. Current bivalent and quadrivalent HPV vaccines target two HR types (HPV16 and 18) responsible for about 70% of cervical cancers [7], whereas the nonavalent vaccine which protects against a wider range of HR-HPV types (HPV16/18/31/33/45/52/58), is estimated to prevent up to 90% of cervical cancers in women from the general population [7, 8]. These potential benefits have seldom been estimated among WLHIV, particularly in sub-Saharan Africa. An improved understanding of HPV type distribution associated with histological lesions in this population is needed to guide HPV vaccine programme decisions.

We conducted a large prospective study of cervical cancer screening in a cohort of women living with HIV-1 in Burkina Faso and South Africa (*HARP—HPV in Africa Research Partnership*). In this paper, we describe (i) the prevalence, persistence, incidence and genotype distribution of HPV, and (ii) their association with prevalent and incident CIN grade 2 and higher (CIN2+).

## Materials and methods

### Study population

Participants were recruited from the HIV outpatient clinic of the University Teaching Hospital of Ouagadougou, Burkina Faso (BF), and HIV treatment centres from inner city Johannesburg South Africa (SA), from December 2011 to October 2012. Inclusion criteria were being HIV-1 seropositive, aged 25–50 years and resident in the city. Exclusion criteria were history of prior treatment for cervical cancer, previous hysterectomy, and being pregnant or less than 8 weeks postpartum. Enrolment was stratified in a 2:1 ratio of ART-users:ART-naïve. Written informed consent was obtained at the screening visit when eligibility for the study was assessed. Participants were followed-up every 6 months for CD4+ T-lymphocytes cell count and up to Month 18 (endline) when procedures similar to baseline were repeated.

### Specimen collection

A venous blood sample was collected to confirm HIV-1 serostatus if necessary, and to obtain CD4+ cell counts. Cervical samples were collected by a nurse-midwife including a Digene cervical sampler at enrolment (baseline) and *care*HPV cervical sampler at endline (both Qiagen, Gaithesburg, MD) for HPV-DNA testing and genotyping. All women were screened for abnormal cervical lesions using cervical smear for cytology by Papanicolaou staining and visual inspection using acetic acid and Lugol’s iodine (VIA/VILI), and referred for colposcopy performed by trained colposcopists. Systematic 4-quadrant cervical biopsy, including directed biopsy of any suspicious lesions, was performed for participants who had abnormalities detected on cytology, VIA/VILI or colposcopy, or who were HR-HPV DNA positive. The same genital sampling and examination procedures were repeated at a scheduled follow-up visit at Month 18.

### Laboratory testing

HIV infection was diagnosed according to national guidelines for each country [9, 10]. Measurement of CD4+ cell count was performed using FACScount (Becton-Dickinson, NJ) in both countries. HPV-DNA genotyping of all samples was performed at the virology laboratory of the Gui de Chauliac Teaching Hospital, University of Montpellier, France, using the INNO-LiPA HPV genotyping Extra^®^ assay (Fujirebio, Courtaboeuf, France) with external quality assurance [11]. HR-HPV types were defined using the current International Agency for Research on Cancer (IARC) classification [12] to include ‘carcinogenic to humans’ (HPV16/18/31/33/35/39/45/51/52/56/58/59) and ‘probable carcinogenic’ (HPV68). The ‘possible carcinogenic’ types (HPV26/53/66/69/70/73/82) and other known low-risk types (HPV6/11/40/43/44/54/71/74) were considered as low-risk (LR-HPV).

Cervical biopsies were processed at the local pathology laboratories in Ouagaoudou and Johannesburg and read using the 3-tier CIN classification system [13]. Histology was classified as “negative” (≤CIN1) or “positive” (CIN2+) based on the highest reading across all findings from the 4-quadrant biopsies and endocervical curettage, if indicated. All histological slides from women with a local diagnosis of CIN2+ and approximately 10% of slides from women with normal or CIN1 histological findings were reviewed by the HARP Endpoint Committee of five pathologists for final classification, after baselineand endline rounds.

### Statistical analysis

HR-HPV genotype-specific persistence was defined as being positive for the same HR-HPV type at baseline and endline visits. Any type clearance was defined as being positive for a specific HR type at baseline and negative for that type at endline visit. Cumulative incidence of any HR-HPV was defined as the proportion of women who were negative for a specific type at baseline and positive for that type at endline visit.

For associations of prevalent and incident CIN2+ with individual HR-HPV types, logistic regression was used to estimate odds ratios (ORs) and 95% confidence intervals (CI). Multivariable analyses adjusted for site and the socio-demographic and behavioural factors which were independently associated in univariate analyses (p<0.10) with CIN2+ for each country, as described in [14], were conducted. Data were analysed using Stata version 14 (Stata Statistical Software, College Station. TX: Stata Corporation).

This study received ethical approval from the Ministry of Health in Burkina Faso (no. 2012-12-089), the University of the Witwatersrand in South Africa (no. 110707), and the London School of Hygiene and Tropical Medicine in the UK (no. 7400).

## Results

### Study population

A full description of study participants has been published elsewhere [14]. Of 1479 women screened, 1238 were enrolled (BF: 615; SA: 623). The median age of participants was 36 (interquartile range [IQR], 31–42) years in BF and 34 (IQR, 30–40) years in SA. About half (49.7%) of SA participants had ever had a Pap smear, and a fifth (20.8%) of BF participants had ever had a VIA/VILI examination, the primary cervical cancer screening modality in each country respectively. At enrolment, 422 (68.6%) participants were on ART in BF and 406 (65.2%) in SA. In BF, the median CD4+ count was 417 (IQR, 315–606) cells/mm^3^ among ART-naive participants and 446 (IQR, 309–600) cells/mm^3^ among ART users. In SA, the median CD4+ count was 448 (IQR, 353–614) cells/mm^3^ among ART-naive and 420 (IQR, 279–567) cells/mm^3^ among ART users.

### Prevalence of HPV and association with CIN2+ at enrolment

Of the 1238 participants enrolled, 1215 (98.1%) had valid HPV genotyping results (BF: 96.6%; SA: 99.7%) and 998 (82.1%) were positive for any HPV (BF: 75.3%; SA: 88.7%). The proportion of multiple HR-HPV genotypes was lower in BF (BF: 41.9% vs. SA: 55.2%; p<0.001; Table 1). HPV52 was the most prevalent type in both countries (BF: 20.4%; SA: 24.2%). HPV16/18 were detected in 15.0% of participants in BF vs. 29.0% in SA (p<0.001). Among those without HPV16/18 co-infection, the proportion infected by any of the additional HR types included in the nonavalent (9v) vaccine (HPV31/33/45/52/58) was 30.1% in BF and 36.7% in SA (p<0.001). The prevalence of any of the non-vaccine HR types (HPV35/39/51/56/59/68) was 35.5% in BF and 49.1% in SA (p<0.001) irrespective of co-infection with any of the vaccine types, and was 14.0% in BF and 13.4% in SA among those not co-infected by any vaccine types.

**Table 1:** HR-HPV infection at baseline and endline follow-up among Women Living with HIV (WLHIV) in Burkina Faso (BF) and South Africa (SA).

	HR-HPV Prevalence		HR-HPV Incidence				HR-HPV Persistence			
	BF N = 594 n (%)	SA N = 621 n (%)	BF		SA		BF		SA	
			At risk^a^ N	Incidence n (%)	At risk^a^ N	Incidence n (%)	Positive at M0 N	Persistence n (%)	Positive at M0 N	Persistence n (%)
**Any HR type**^b^	351 (59.1)	491 (79.1)	476	228 (47.9)	446	220 (49.3)	**270**	139 (51.5)	340	152 (44.7)
***Any alpha-9***	264 (44.4)	392 (63.1)	476	152 (31.9)	446	154 (34.5)	**198**	98 (49.5)	**260**	99 (38.1)
**HPV16**	51 (8.6)	119 (19.2)	445	43 (9.7)	377	49 (13.0)	**31**	17 (54.8)	**69**	23 (33.3)
**HPV31**	47 (7.9)	65 (10.5)	445	44 (9.9)	404	22 (5.5)	**31**	19 (61.3)	**42**	6 (14.3)
**HPV33**	19 (3.2)	51 (8.2)	464	8 (1.7)	419	17 (4.1)	**12**	3 (25.0)	**27**	4 (14.8)
**HPV35**	62 (10.4)	103 (16.6)	429	24 (5.6)	385	34 (8.8)	**47**	18 (38.3)	**61**	26 (42.6)
**HPV52**	121 (20.4)	150 (24.2)	378	48 (12.7)	337	56 (16.6)	**98**	37 (37.8)	**109**	39 (35.8)
**HPV58**	27 (4.6)	55 (8.9)	458	15 (3.3)	417	14 (3.4)	**18**	13 (72.2)	**29**	11 (37.9)
***Any alpha-7***	117 (19.7)	198 (31.9)	476	117 (24.6)	446	122 (27.4)	**89**	37 (41.6)	**145**	46 (31.7)
**HPV18**	42 (7.1)	90 (14.5)	445	29 (6.5)	378	18 (4.8)	**31**	17 (54.8)	**68**	24 (35.3)
**HPV39**	43 (7.2)	50 (8.1)	442	24 (5.4)	408	25 (6.1)	**34**	7 (20.6)	**38**	8 (21.1)
**HPV45**	26 (4.4)	48 (7.7)	457	12 (2.6)	415	22 (5.3)	**19**	11 (57.9)	**31**	11 (35.5)
**HPV59**	6 (1.0)	12 (1.9)	473	4 (0.9)	436	5 (1.2)	**3**	0 (0.0)	**10**	2 (20.0)
**HPV68**	23 (3.9)	35 (5.6)	457	24 (5.3)	424	28 (6.6)	**19**	3 (15.8)	**22**	4 (18.2)
***Alpha-5 and -6***										
**HPV51**	70 (11.8)	97 (15.6)	423	30 (7.1)	378	22 (5.8)	**53**	15 (28.3)	**68**	18 (26.5)
**HPV56**	25 (4.2)	60 (9.7)	456	45 (9.9)	408	16 (3.9)	**20**	11 (55.0)	**38**	9 (23.7)
***Combinations***										
**HPV16/18**	89 (15.0)	180 (29.0)	473	69 (14.6)	422	62 (14.7)	**59**	33 (55.9)	**113**	45 (39.8)
**Other 9vHPV**^c^	179 (30.1)	228 (36.7)	473	93 (19.7)	422	101 (23.9)	**155**	70 (42.5)	**198**	61 (30.8)
**Any 9vHPV HR**^d^	268 (45.1)	408 (65.7)	476	162 (34.0)	446	163 (36.6)	**200**	103 (51.5)	**275**	106 (38.6)
**Non-Vaccine**^e^	83 (14.0)	83 (13.4)	473	66 (14.0)	422	57 (13.5)	**112**	36 (32.1)	**141**	46 (32.6)
**Multiple HR**	147/351 (41.9)	271/491 (55.2)	228	81 (35.5)	220	79 (35.9)	**270**	27/139 (19.4)	340	27/152 (17.8)
***Low risk types***										
**HPV6**	34 (5.7)	33 (5.3)	450	22 (4.9)	422	13 (3.1)	**26**	6 (23.1)	**24**	2 (8.3)
**HPV11**	9 (1.5)	33 (5.3)	470	3 (0.6)	420	15 (3.6)	**6**	1 (16.7)	**26**	4 (15.4)

^**a**^negative for that HPV type at enrolment (3 participants in BF with positive 16 *AND* 18 at enrolment, and 24 in SA; no participant was infected by ALL nonavalent HR types or ALL non vaccine types;

^**b**^Any HR-HPV type prevalence defined as positive for at least one HR type at baseline; any HR incidence defined as incident infection from at least one HR type among those at risk for any HR infection (no participant was infected by all HR types at baseline); persistence defined as persistence of at least one HR type among those positive for any HR type at baseline;

^**c**^Other 9vHPV = Positive for any of HPV31/33/45/52/ 58 in absence of HPV16/18;

^**d**^9vHPV HR = Positive for any of HPV16/18/31/33/45/52/58;

^**e**^Non Vaccine = Positive for any of HPV35/39/51/56/59/68 in absence of any vaccine type.

**Fig 1:**
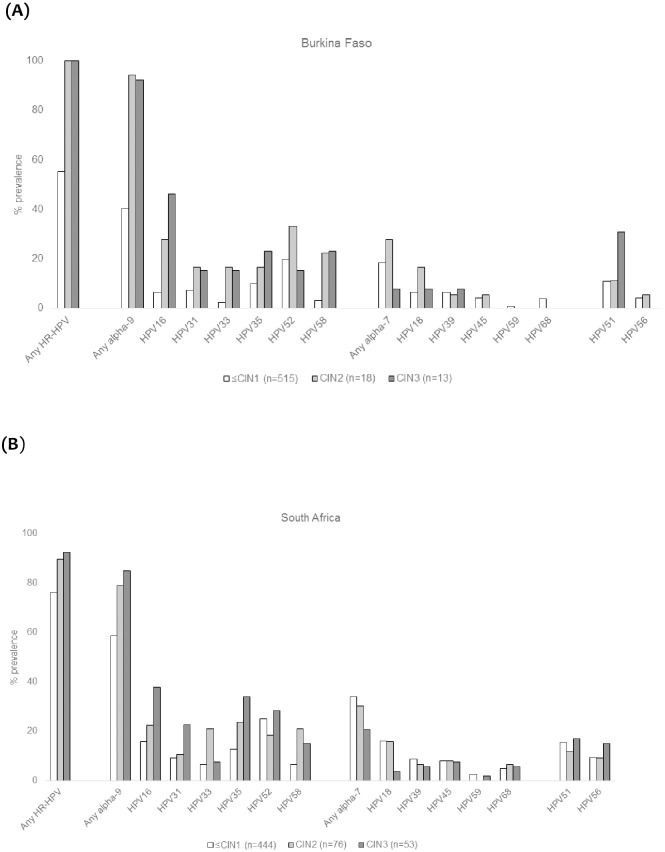
(A) HR-HPV prevalence by CIN grade among 546 women living with HIVin Burkina Faso. (B) HR-HPV prevalence by CIN grade among 573 women living with HIV in South Africa.

Among women with both histology and HPV results (BF: 546; SA: 573), the prevalence of HR-HPV was higher among those with higher CIN grades in both settings (BF: 48.4% in CIN normal, 71.8% in CIN1, 100% in CIN2 and 100% in CIN3+, p<0.001; SA: 72.4% in CIN normal, 81.4% in CIN1, 89.5% in CIN2 and 92.5% in CIN3+, p<0.001;) (Fig 1(A) and 1(B)). The prevalence of any HR-HPV genotypes included in the bi/quadrivalent and nonavalent HPV vaccines was 45.2% and 90.3% among women with CIN2+ in BF, respectively, and 37.2% and 79.8% in SA, respectively. In the absence of any vaccine types, the prevalence of any non-vaccine types was 9.7% (3/31) in BF and 10.9% (14/129) in SA.

**Fig 2:**
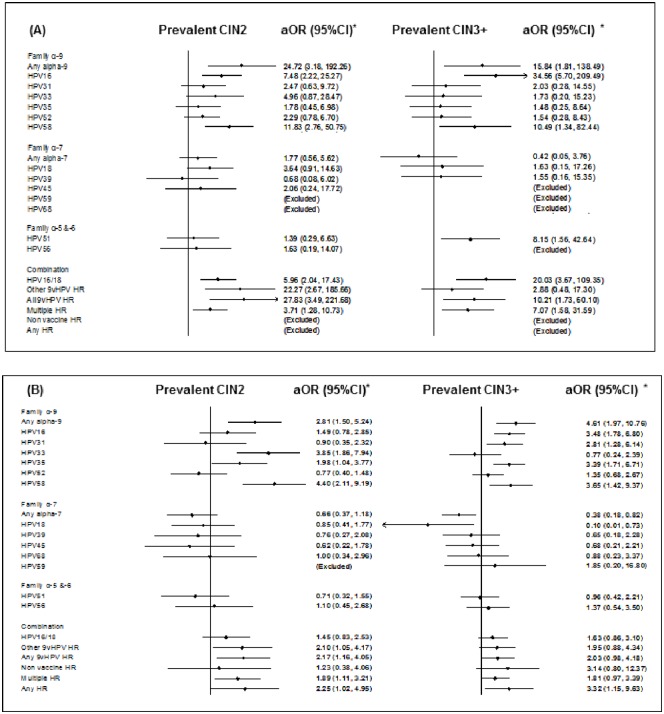
(A) Association of HR-HPV prevalence with prevalent CIN2 and CIN3+ among 546 women living with HIV in Burkina Faso. (B) Association of HR-HPV prevalence with prevalent CIN2 and CIN3+ among 573 women living with HIV in South Africa.

In both countries, HPV58 was the genotype most strongly associated with CIN2 compared to ≤CIN1 cases (adjusted Odds Ratio [aOR] = 5.40, 95%CI: 2.77–10.53), while types most strongly associated with CIN3+ were HPV16 in BF (aOR = 34.56, 95%CI: 5.70–209.49) and HPV58 in SA (aOR = 3.65, 95%CI: 1.42–9.37) (Fig 2(A) and 2(B) and S1 Table). The prevalence of HPV58 was 22.1% and 21.1% among women with CIN2 in BF and SA respectively, and 23.1% and 15.1% among women with CIN3+.

The prevalence of CIN2+ was significantly higher among those positive for HPV16/18 compared to those who were negative for both types in BF (17.7% vs. 3.6%; aOR = 7.90, 95%CI: 3.23–19.33) and in SA (29.1% vs. 19.9%, aOR = 1.49, 95%CI: 0.95–2.34), although the latter was not statistically significant (S1 Table). Among 875 women without co-infection of either HPV16 or 18, the prevalence of CIN2+ was higher among women positive for any of the additional HR-HPV types targeted by the nonavalent vaccine (31/33/45/52/58) compared to women negative for all of these types in both countries (BF: 8.8% vs. 1.0%; SA: 25.2% vs. 13.7%; aOR = 2.78, 95%CI: 1.67–4.64 for both countries combined). The prevalence of CIN2+ was similar among women who were positive or negative for any of the non-vaccine HR-HPV types. However, among 498 women without infection with vaccine types (BF: 308, SA: 190), the prevalence of CIN2+ was higher among women positive for any of the non-vaccine types compared to women who were HR-HPV negative (11.1% vs. 3.4%; aOR = 3.35, 95%CI: 1.36–8.26).

### Incidence and persistence of HR-HPV

Of the 963 women without CIN2+ at enrolment who returned at follow-up M18 visit (median of actual follow-up 16 months [IQR, 15.6–16.8]), genotyping data at both visits was available for 922 (95.7%) women. The proportion of women with an incident HR-HPV infection was similar in both sites (BF: 47.9% [228/476] vs. SA: 49.3% [220/446]; p = 0.67). Type-specific incident infection was highest for HPV52 in both countries (BF: 12.7%; SA: 16.6%). Incident infection of HPV16/18 was observed in 14.6% of women in BF and 14.7% in SA. In the absence of HPV16/18 co-infection, incident infection of any of the additional HR-HPV types of the nonavalent vaccine was observed in 19.7% of women in BF and 23.9% in SA; incident infection of non-vaccine HR-HPV types in the absence of vaccine type co-infection was observed in 14.0% in BF and 13.5% in SA (Table 1).

Persistence was highest for HPV58 (72.2%) in BF and HPV35 (42.6%) in SA. Forty-nine women (18.2%) in BF and 80 (23.5%) in SA had at least one persistent and one cleared infection during the 16 months follow-up period (Table 1).

### Association of incident CIN2+ with HR-HPV persistence

Among 922 women with genotyping data at both visits, concomitant histology results were available for 780 (84.6%) women (BF: 405; SA: 375). Cumulative incidence of CIN2+ was higher among those with HR-HPV persistence compared to those who cleared or who were HR-HPV negative at baseline in both settings (BF: 3.5% vs. 0.3%; SA: 13.4% vs. 2.0%; both countries combined aOR = 7.90, 95%CI: 3.11–20.07, Table 2).

**Table 2:** Risk of incident CIN2+ according to HR-HPV infection status over 16 months among 405 Women Living with HIV (WLHIV) without CIN2+ at enrolment in Burkina Faso and 375 WLHIV in South Africa.

	Burkina Faso						South Africa						Sites combined						
	Always negative or incident infection		Cleared infection or type swap*		Persistent infection		Always negative or incident infection		Cleared infection or type swap*		Persistent infection		Always negative or incident infection		Cleared infection or type swap*		Persistent infection		aOR (95%CI) ^d^^,^^e^
	N^a^	n (%)	N^b^	n (%)	N^c^	n (%)	N^a^	n (%)	N^b^	n (%)	N^c^	n (%)	N^a^	n (%)	N^b^	n (%)	N^c^	n (%)	
**Any HR type**^f^	**177**	**0 (0.0)**	**113**	**1 (0.9)**	**115**	**4 (3.5)**	**91**	**1 (1.1)**	**157**	**4 (2.6)**	**127**	**17 (13.4)**	**268**	**1 (0.4)**	**270**	**5 (1.9)**	**242**	**21 (8.7)**	**7.90 (3.11–20.07)**
*Any alpha-9*	236	1 (0.4)	85	1 (1.2)	84	3 (3.6)	158	3 (1.9)	135	5 (3.7)	82	14 (17.1)	394	4 (1.0)	220	6 (2.7)	166	17 (10.2)	**6.91 (3.05–15.63)**
HPV16	380	4 (1.1)	10	0 (0.0)	15	1 (6.7)	321	16 (5.0)	39	3 (7.7)	15	3 (20.0)	701	20 (2.9)	49	3 (6.1)	30	4 (13.3)	**4.75 (1.47–15.36)**
HPV31	379	5 (1.3)	11	0 (0.0)	15	0 (0.0)	343	21 (6.1)	28	1 (3.6)	4	0 (0.0)	722	26 (3.6)	39	1 (2.6)	19	0 (0.0)	-
HPV33	395	4 (1.0)	7	1 (14.3)	3	0 (0.0)	354	21 (5.9)	19	1 (5.3)	2	0 (0.0)	749	25 (3.3)	26	2 (7.7)	5	0 (0.0)	-
HPV35	360	4 (1.1)	28	0 (0.0)	17	1 (5.9)	327	18 (5.5)	28	0 (0.0)	20	4 (20.0)	687	22 (3.2)	56	0 (0.0)	37	5 (13.5)	**4.72 (1.62–13.71)**
HPV52	320	3 (0.9)	52	1 (1.9)	33	1 (3.0)	278	17 (6.1)	60	2 (3.3)	37	3 (8.1)	598	20 (3.3)	112	3 (2.7)	70	4 (5.7)	1.67 (0.55–5.05)
HPV58	392	5 (1.3)	3	0 (0.0)	10	0 (0.0)	349	18 (5.2)	17	0 (0.0)	9	4 (44.4)	741	23 (3.1)	20	0 (0.0)	19	4 (21.1)	**9.56 (2.77–32.98)**
*Any alpha-7*	333	4 (1.2)	42	0 (0.0)	30	1 (3.3)	249	11 (4.4)	86	5 (5.8)	40	6 (15.0)	582	15 (2.6)	128	5 (3.9)	70	7 (10.0)	**3.34 (1.33–8.37)**
HPV18	381	4 (1.1)	12	0 (0.0)	12	1 (8.3)	318	15 (4.7)	38	3 (7.9)	19	4 (21.1)	699	19 (2.7)	50	3 (6.0)	31	5 (16.1)	**5.39 (1.83–15.82)**
HPV39	378	5 (1.3)	21	0 (0.0)	6	0 (0.0)	340	20 (5.9)	28	2 (7.1)	7	0 (0.0)	718	25 (3.5)	49	2 (4.1)	13	0 (0.0)	-
HPV45	388	5 (1.3)	7	0 (0.0)	10	0 (0.0)	348	20 (5.8)	16	1 (6.3)	11	1 (9.1)	736	25 (3.4)	23	1 (4.4)	21	1 (4.8)	1.28 (0.16–10.13)
HPV59	404	5 (1.2)	1	0 (0.0)	0	0 (0.0)	365	21 (5.8)	8	0 (0.0)	2	1 (50.0)	769	26 (3.4)	9	0 (0.0)	2	1 (50.0)	16.03 (0.96–268.33)
HPV68	388	5 (1.3)	14	0 (0.0)	3	0 (0.0)	357	21 (5.9)	14	0 (0.0)	4	1 (25.0)	745	26 (3.5)	28	0 (0.0)	7	1 (14.3)	4.15 (0.45–38.02)
HPV51	364	5 (1.4)	33	0 (0.0)	8	0 (0.0)	319	19 (6.0)	43	2 (4.7)	13	1 (7.7)	683	24 (3.5)	76	2 (2.6)	21	1 (4.8)	1.21 (0.15–9.53)
HPV56	387	4 (1.0)	9	0 (0.0)	9	1 (11.1)	340	20 (5.9)	27	0 (0.0)	8	2 (25.0)	727	24 (3.3)	36	0 (0.0)	17	3 (17.7)	**6.77 (1.69–27.04)**
*Combination*																			
HPV16/18	358	3 (0.8)	21	0 (0.0)	26	2 (7.7)	284	12 (4.2)	58	4 (6.9)	33	6 (18.2)	642	15 (2.3)	79	4 (5.1)	59	8 (13.6)	**5.25 (2.14–12.91)**
9vHPV 5 HR^g^	250	0 (0.0)	70	2 (2.9)	59	1 (1.7)	173	8 (4.6)	113	2 (1.8)	56	6 (10.7)	423	8 (1.9)	183	4 (2.2)	115	7 (6.1)	**3.23 (1.23–8.54)**
Any 9vHPV	240	0 (0.0)	80	2 (2.5)	85	3 (3.5)	145	5 (3.5)	141	5 (3.6)	89	12 (13.5)	385	5 (1.3)	221	7 (3.2)	174	15 (8.6)	**4.46 (2.02–9.86)**
Non-Vaccine^h^	224	1 (0.5)	66	0 (0.0)	30	1 (3.3)	164	3 (1.8)	84	2 (2.4)	38	5 (13.2)	388	4 (1.0)	150	2 (1.3)	68	6 (8.8)	**7.87 2.40–25.81)**
**Selected low risk types**																			
HPV6	382	5 (1.3)	19	0 (0.0)	4	0 (0.0)	354	22 (6.2)	19	0 (0.0)	2	0 (0.0)	736	27 (3.7)	38	0 (0.0)	6	0 (0.0)	-
HPV11	401	5 (1.3)	3	0 (0.0)	1	0 (0.0)	354	21 (5.9)	18	1 (5.6)	3	0 (0.0)	755	26 (3.4)	21	1 (4.8)	4	0 (0.0)	-

^**a**^Total number of women who were negative throughout follow-up or who had incident type specific HPV infection;

^**b**^total number of women who had a cleared type specific infection or acquired a new type (type swap);

^**c**^total number of women who had type specific persistent infection;

^d^OR for incident CIN2+ among those with HR-HPV persistence compared to all other participants at endline (includes those that were negative for that type or those that developed incident infection with that type during follow-up;

^e^adjusted for site and ART status at enrolment

^f^Any HR type persistence is defined as those that had at least one HR type persistence; clearance or incidence among those who did not persist; and negative at baseline includes those who were negative for all HR types at baseline;

^g^Persistence of any of HPV31/33/45/52/58 among those without persistent HPV16/18;

^h^Persistence of any of HPV35/39/51/56/59/68 among those without persistent HPV16/18/31/33/45/52/58;

*Type swap is defined as clearance of one genotype and acquisition of a different genotype

CIN2+ incidence was found to be significantly associated with the persistence of any members of the alpha-9 HPV (HPV16-related) and any alpha-7 HPV (HPV-18 related) genotypes in both countries **(**Table 2). In particular, CIN2+ incidence was higher among women with persistent HPV16/18 compared to those who cleared either or who were negative for both at baseline (BF: 7.7% vs. 0.8%; SA: 18.2% vs. 4.7%; both sites combined: aOR = 5.25, 95%CI: 2.14–12.91).

Among 721 women without persistent HPV16 or 18, CIN2+ incidence was higher among those with persistence of any of the additional 5 HR-HPV included in the nonavalent (both sites combined: 6.1% vs. 2.0% aOR = 3.23, 95%CI: 1.23–8.54). In the absence of co-persistence by any of the vaccine types (606 women), CIN2+ incidence was higher among women with persistent non-vaccine types compared to those who cleared or who were HR-HPV negative at baseline in both settings (8.1% vs. 1.1%; aOR = 7.87, 95%CI: 2.40–25.81).

### Association of HIV-related factors with HR-HPV prevalence at baseline

The prevalence of HR-HPV types was similar among ART users and ART-naive participants at baseline in BF, whilst in SA, the prevalence of HPV16/18 was higher among ART-naïve participants (S2 and S3 Tables). Among ART users in BF, the prevalence of any nonavalent HR-HPV in the absence of HPV16/18 was higher among women with low CD4+ count (<200 cells/mm^3^) compared to those with high CD4+ count (>500 cells/mm^3^) and this association was significant among ART users only, however no association was observed for HPV16/18 or the non-vaccine types. In SA, only the prevalence of non-vaccine HR-HPV was higher among ART-taking women with low CD4+ count (S3 Table).

## Discussion

Women living with HIV (WLHIV) in Burkina Faso and South Africa have a high prevalence, incidence and persistence of HR-HPV and correspondingly a high prevalence and incidence of cervical neoplasia. The high rates of HR-HPV found in both countries are similar to other studies in Sub Saharan Africa [1, 2, 15, 16]. The increased risk in the prevalence of HR-HPV and CIN2+ among women in South Africa compared to Burkina Faso may be explained by the less well controlled HIV disease (in terms of lower rates of ART adherence and HIV viral suppression) found among women in South Africa, as we reported elsewhere [14], as well as by other cofactors for HR-HPV and CIN2+, such as greater frequency of contraceptive use and smoking and higher prevalence of mucosal STIsobserved in this and other cohort of WLHIV [17–25].

While HR-HPV prevalence was higher in South Africa at baseline, HR-HPV persistence was higher among women in Burkina Faso. This may be due to the fact that estimates of HR-HPV persistence were restricted to women without evidence of CIN2+ at baseline, who underwent treatment. As there was a higher prevalence of CIN2+ at baseline in South Africa, their exclusion from the follow-up analysis potentially reduced the overall proportion of women with persistent infection at endline. By contrast, because of the lower prevalence of CIN2+ in Burkina Faso at baseline, fewer women were excluded in the follow-up analysis, which potentially allowed a greater number of persistent HR-HPV cases to accrue during the follow-up period. In addition, we cannot exclude the possibility that the 4-quadrant biopsy may have helped clear HR-HPV from the cervical epithelium, as a greater proportion of women in South Africa underwent a 4-quadrant biopsy.

HPV52 was the type most frequently detected overall in both countries, but HPV58 was the type most strongly associated with cervical lesions, and one of the most likely to persist in both countries. In a meta-analysis describing HPV type distribution among 5578 WLHIV from 20 studies including 5 from African countries, HPV16, 18 and 58 were the most prevalent among those with cytological high-grade squamous intraepithelial lesions (HSIL+) [3, 26]. Additionally, WLHIV with HSIL+ were less likely to harbour HPV16 compared to those with HSIL in the general population, and more likely to be infected with HPV18/33/51/52/58, suggesting that the HPV16/18 based bi/quadrivalent vaccines may have a lower impact on cervical cancer rates in settings where HIV prevalence is high [27]. The high prevalence of types other than HPV16 and HPV18 among WLHIV in this study is similar to what has been reported elsewhere [6]. While we found that up to 45% of CIN2+ in BF and 37% of CIN2+ in SA could be prevented by the HPV16/18-targeting vaccines, up to 90% of CIN2+ in BF and 80% of CIN2+ in SA could be prevented by the nonavalent vaccine, as predicted from studies in the general population [8, 28].

We found that up to 45% of CIN2/3 in BF and 37% of CIN2/3 in SA could be prevented by the HPV16/18-targeting vaccines, and that a further 45% of CIN2/3 in BF and 43% of CIN2/3 in SA could be prevented by the additional 5 types (HPV31/33/45/51/52) contained in the nonavalent vaccine. However, not all CIN2/3 develop into invasive cervical cancer (ICC), and not all HPV types found in CIN2 have the same propensity to evolve towards ICC. Clifford et al, in their systematic review comparing the HPV type distribution in ICC biopsy and cervical specimens of 770 HIV-positive women from 21 studies in 12 African countries [29], report a higher pooled prevalence of HPV16/18 (61.7%) than found in this study, whilst the prevalence of the other nonavalent vaccine types was 11.2%, lower than what we report in our study. These findings indicate that the relative contribution of HPV16/18 increases with increasing lesion grade, whilst the contribution of the additional nonavalent vaccine still represents a significant preventable fraction of ICC.

Our study also shows that the persistence of any of the bi/quadrivalent and nonavalent HR-HPV types is strongly associated with the incidence of cervical lesions among WLHIV, as is the case for HIV uninfected women [30]. Moreover, our study found a significant proportion of women had an incident infection with any of the nonavalent vaccine types (35%) as well as incident infection of multiple HR-HPV types over the 16-month follow-up period. This emphasises the potential impact of vaccination in this population using an HPV vaccine with a broader-range protection potential, given that these types are also likely to persist in this population [31].

A smaller but non-negligible proportion (11%) of women with CIN2+ were exclusively infected by non-vaccine types at enrolment. In addition, the persistence of non-vaccine types in the absence of current vaccine types, was also strongly associated with incident CIN2+, and this was largely driven by persistence of HPV35 and 56. A large retrospective cross-sectional study using histologically conformed ICC collected from 38 countries worldwide reported that 91% of cases were positive for any of the current nonavalent vaccine types in addition to HPV35 [7]. Until further generation vaccines incorporate a wider range of HPV types, cervical screening will remain important among WLHIV where non-vaccine types are prevalent.

ART-naïve women were at increased risk of HPV16 or 18 infection in South Africa at baseline. Among ART users, a decrease in CD4+ count was also associated with an increased risk of any of the nonavalent other than HPV16/18 and the non-vaccine type prevalence in Burkina Faso, and of the non-vaccine types in South Africa. However, there was no reduction in HPV16 or 18 prevalence with increasing CD4+ counts in either country. This is consistent with previous reports suggesting that HPV16 might be more weakly associated with immune suppression than other HR types due to its potential to evade the host immune response, thus explaining its greater contribution to high-grade lesions and cancer in immunocompetent women [32]. However, these associations may also be reflective of the overall increased risk of any HR-HPV prevalence among those with low CD4+, as we have previously reported elsewhere [14].

Given that the bi/quadrivalent and nonavalent types are the most important contributors to cervical lesions in this population, maintaining a stable high CD4+ count is necessary to reduce the risks of HR-HPV persistence and cervical lesion development. This should be achieved by early initiation of ART before CD4+ counts decline and maintaining good HIV-1 virological control thereafter. In addition, HPV vaccination could be offered to these women before CD4+ counts decline.

Limitations of this study include the absence of HPV genotyping from the biopsies, which preclude accurate attribution of CIN to particular genotypes. Furthermore, our definition of cumulative HR-HPV incidence over 16 months is at the same time crude and of limited duration to assess the actual roles of HR-HPV incidence and persistence on CIN2+ incidence. Other studies have used shorter time intervals for defining HR-HPV incidence [33] because of the transient nature of HR-HPV infection. Furthermore, the study could not rule out type-specific clearance and reinfection when estimating persistence during the 16-months interval between HPV testing. The evaluation of HPV at two time points only did not allow precise estimation of the duration of infections. Longer duration of follow-up would have allowed to accrue a larger number of incident CIN2+ cases and more robustly assess the role of incident HR-HPV on CIN development over sufficient follow-up time. A key strength of this study, however, is the availability of a large proportion of women with verified histological endpoints and concurrent genotyping data. In addition, the HARP study was undertaken in two African countries with very different HIV epidemics and possibly different burdens of HPV infection and cervical cancer, but the similarity of data allows the findings to be extended to a range of countries and settings in sub-Saharan Africa.

In conclusion, we confirm that HR-HPV infection and cervical lesions are very common among WLHIV in Africa, and that a broader range of genotypes are potentially associated with CIN2+ development. While currently available bi/quadrivalent vaccines could prevent up to 45% of treatable precursor lesions, the nonavalent vaccine has the potential to prevent up to 90% of cases in WLHIV. HPV vaccination could reduce the incidence of HR-HPV related disease among WLHIV in addition to contributing cost saving to current screening and treatment programmes, but this would require a formal health economic evaluation.

## Supporting information

S1 Table Association of HPV type prevalence with prevalent CIN2 and CIN3+ among 546 women living with HIV in Burkina Faso and 573 in South Africa. (DOCX)

S2 Table Association of HR-HPV type prevalence with ART and CD4+ count at enrolment among 570 women living with HIV in Burkina Faso. (DOCX)

S3 Table Association of HR-HPV type prevalence with ART and CD4+ count at enrolment among 613 women living with HIV in South Africa. (DOCX)
