# Supplementary material for: Associations of Human Papillomavirus (HPV) genotypes with high-grade cervical neoplasia (CIN2+) in a cohort of women living with HIV in Burkina Faso and South Africa
Source: PLoS One. 2017 Mar 23;12(3):e0174117. doi: 10.1371/journal.pone.0174117 (PMC5363860; doi:10.1371/journal.pone.0174117)
Supplement: S1 Table — (DOCX) [file pone.0174117.s001.docx]

**S1 Table.** Association of HPV type prevalence with prevalent CIN2 and CIN3+ among 546 women living with HIV in Burkina Faso and 573 in South Africa

|  | **Burkina Faso** | | | | | | | **South Africa** | | | | | | |
| --- | --- | --- | --- | --- | --- | --- | --- | --- | --- | --- | --- | --- | --- | --- |
|  | **CIN2**  **(n=18)** | | | **CIN3+**  **(n=13)** | | | **CIN2+**  **(n-31)** | **CIN2**  **(n=76)** | | | **CIN3+**  **(n=53)** | | | **CIN2+**  **(n=129)** |
|  | **HR negative** | **HR positive** | **aOR (95% CI** **)^a^** | **HR negative** | **HR positive** | **aOR (95% CI** **)^a^** | **aOR (95% CI** **)^a^** | **HR negative** | **HR positive** | **aOR (95% CI** **)^b^** | **HR negative** | **HR positive** | **aOR (95% CI** **)^b^** | **aOR (95% CI** **)^b^** |
|  | **n (%)** | **n (%)** |  | **n (%)** | **n (%)** |  |  | **n (%)** | **n (%)** |  | **n (%)** | **n (%)** |  |  |
| **Any HR-HPV** | **0 (0.0)** | **18 (6.0)** | **(-)** | **0 (0.0)** | **13 (4.4)** | **(-)** | **(-)** | **8 (7.0)** | **68 (16.8)** | **2.25 (1.02-4.95)** | **4 (3.6)** | **49 (12.7)** | **3.32 (1.15-9.63)** | **2.64 (1.37-5.07)** |
|  |  |  |  |  |  |  |  |  |  |  |  |  |  |  |
| *Any alpha-9* | 1 (0.3) | 17 (7.6) | **24.72 (3.18-192.26)** | 1 (0.3) | 12 (5.5) | **15.84 (1.81-138.49)** | **19.89 (4.51-87.84)** | 16 (8.0) | 60 (18.8) | **2.81 (1.50-5.24))** | 8 (4.2) | 45 (14.8) | **4.61 (1.97-10.76)** | **3.36 (1.99-5.66)** |
| HPV16 | 13 (2.6) | 5 (13.2) | **7.48 (2.22-25.27)** | 7 (1.4) | 6 (15.4) | **34.56 (5.70-209.49)** | **10.82 (4.03-29.06)** | 59 (13.6) | 17 (19.5) | 1.49 (0.78-2.85) | 33 (8.1) | 20 (22.2) | **3.48 (1.78-6.80)** | **2.06 (1.25-3.39)** |
| HPV31 | 15 (3.0) | 3 (7.5) | 2.47 (0.63-9.72) | 11 (2.3) | 2 (5.1) | 2.03 (0.28-14.55) | 2.27 (0.71-7.26) | 68 (14.4) | 8 (16.3) | 0.90 (0.35-2.32) | 41 (9.2) | 12 (22.6) | **2.81 (1.28-6.14)** | 1.63 (0.85-3.11) |
| HPV33 | 15 (2.9) | 3 (20.0) | 4.96 (0.87-28.47) | 11 (2.1) | 2 (14.3) | 1.73 (0.20-15.23) | 3.15 (0.76-13.06) | 60 (12.6) | 16 (35.6) | **3.85 (1.86-7.94)** | 49 (10.6) | 4 (12.1) | 0.77 (0.24-2.39) | **2.46 (1.29-4.70)** |
| HPV35 | 15 (3.1) | 3 (5.6) | 1.78 (0.45-6.98) | 10 (2.1) | 3 (5.6) | 1.48 (0.25-8.64) | 1.55 (0.51-4.70) | 58 (13.0) | 18 (24.0) | **1.98 (1.04-3.77)** | 35 (8.3) | 18 (24.0) | **3.39 (1.71-6.71)** | **2.60 (1.56-4.34)** |
| HPV52 | 12 (2.8) | 6 (5.6) | 2.29 (0.78-6.70) | 11 (2.6) | 2 (1.9) | 1.54 (0.28-8.43) | 1.91 (0.76-4.83) | 62 (15.7) | 14 (11.2) | 0.77 (0.40-1.48) | 38 (10.2) | 15 (11.9) | 1.35 (0.68-2.67) | 0.97 (0.59-1.59) |
| HPV58 | 14 (2.7) | 4 (20.0) | **11.83 (2.76-50.75)** | 10 (2.0) | 3 (15.8) | **10.49 (1.34-82.44)** | **10.74 (3.18-36.33)** | 60 (12.6) | 16 (35.6) | **4.40 (2.11-9.19)** | 45 (9.8) | 8 (21.6) | **3.65 (1.42-9.37)** | **4.13 (2.16-7.88)** |
|  |  |  |  |  |  |  |  |  |  |  |  |  |  |  |
| *Any alpha-7* | 13 (3.0) | 5 (5.1) | 1.77 (0.56-5.62) | 12 (2.8) | 1 (1.1) | 0.42 (0.05-3.76) | 1.19 (0.43-3.27) | 53 (15.3) | 23 (13.2) | 0.66 (0.37-1.18) | 42 (12.5) | 11 (6.8) | **0.38 (0.18-0.82)** | **0.55 (0.34-0.89)** |
| HPV18 | 15 (3.0) | 3 (8.1) | 3.64 (0.91-14.63) | 12 (2.4) | 1 (2.9) | 1.63 (0.15-17.26) | 2.81 (0.83-9.44) | 64 (14.7) | 12 (14.5) | 0.85 (0.41-1.77) | 51 (12.0) | 2 (2.7) | **0.10 (0.01-0.73)** | 0.53 (0.27-1.05) |
| HPV39 | 17 (3.4) | 1 (2.9) | 0.68 (0.08-6.02) | 12 (2.4) | 1 (2.9) | 1.55 (0.16-15.35) | 0.90 (0.18-4.45) | 71 (14.9) | 5 (11.4) | 0.76 (0.27-2.08) | 50 (11.0) | 3 (7.1) | 0.65 (0.18-2.28) | 0.74 (0.33-1.70) |
| HPV45 | 17 (3.3) | 1 (4.6) | 2.06 (0.24-17.72) | 13 (2.6) | 0 (0.0) | (-) | 1.22 (0.15-10.04) | 70 (14.6) | 6 (14.6) | 0.62 (0.22-1.78) | 49 (10.7) | 4 (10.3) | 0.68 (0.21-2.21) | 0.65 (0.28-1.49) |
| HPV59 | 18 (3.4) | 0 (0.0) | (-) | 13 (2.5) | 0 (0.0) | (-) | (-) | 76 (14.9) | 0 (0.0) | (-) | 52 (10.7) | 1 (8.3) | 1.85 (0.20-16.80) | 0.50 (0.06-4.16) |
| HPV68 | 18 (3.5) | 0 (0.0) | (-) | 13 (2.6) | 0 (0.0) | (-) | (-) | 71 (14.4) | 5 (18.5) | 1.00 (0.34-2.96) | 50 (10.6) | 3 (12.0) | 0.88 (0.23-3.37) | 1.00 (0.40-2.50) |
|  |  |  |  |  |  |  |  |  |  |  |  |  |  |  |
| HPV51 | 16 (3.4) | 2 (3.5) | 1.39 (0.29-6.63) | 9 (1.9) | 4 (6.7) | **8.15 (1.56-42.64)** | 2.54 (0.87-7.37) | 67 (15.2) | 9 (11.5) | 0.71 (0.32-1.55) | 44 (10.5) | 9 (11.5) | 0.96 (0.42-2.21) | 0.78 (0.43-1.44) |
| HPV56 | 17 (3.3) | 1 (4.6) | 1.63 (0.19-14.07) | 13 (2.6) | 0 (0.0) | (-) | 0.90 (0.10-7.85) | 69 (14.7) | 7 (14.3) | 1.10 (0.45-2.68) | 45 (10.1) | 8 (16.0) | 1.37 (0.54-3.50) | 1.20 (0.60-2.42) |
|  |  |  |  |  |  |  |  |  |  |  |  |  |  |  |
| *Combinations* |  |  |  |  |  |  |  |  |  |  |  |  |  |  |
| HPV16/18 | 11 (2.4) | 7 (9.7) | **5.96 (2.04-17.43)** | 6 (1.3) | 7 (9.7) | **20.03 (3.67-109.35)** | **7.90 (3.23-19.33)** | 49 (13.0) | 27 (18.8) | 1.45 (0.83-2.53) | 32 (8.9) | 21 (15.2) | 1.63 (0.86-3.10) | 1.49 (0.95-2.34) |
| 9vHPV 5 HR | 1 (0.3) | 10(6.5) | **22.27 (2.67-185.66)** | 2 (0.7) | 4 (2.7) | 2.88 (0.48-17.30) | **12.30 (2.88-52.46)** | 15 (8.4) | 34 (17.3) | **2.10 (1.05-4.17)** | 11 (6.3) | 21 (11.4) | 1.95 (0.88-4.34) | **1.98 (1.15-3.42)** |
| Any 9vHPV 7 HR | 1 (0.3) | 17 (7.5) | **27.83 (3.49-221.68)** | 2 (0.7) | 11 (5.0) | **10.21 (1.73-60.10)** | **16.38 (4.43-60.58)** | 15 (8.4) | 61 (17.9) | **2.17 (1.16-4.05)** | 11 (6.3) | 42 (13.0) | 2.03 (0.98-4.18) | **2.07 (1.26-3.41)** |
| Non Vaccine | 0 (0.0) | 1 (1.3) | **(-)** | 0 (0.0) | 2 (2.6) | (-) | (-) | 8 (7.0) | 7 (10.8) | 1.23 (0.38-4.06) | 4 (3.6) | 7 (10.8) | 3.14 (0.80-12.37) | 1.93 (0.80-4.67) |
| Multiple HR**^c^** | 10 (2.4) | 8 (6.7) | **3.71 (1.28-10.73)** | 6 (1.5) | 7 (5.9) | **7.07 (1.58-31.59)** | **4.26 (1.79-10.13)** | 34 (11.3) | 42 (19.1) | **1.89 (1.11-3.21)** | 22 (7.6) | 31 (14.8) | 1.81 (0.97-3.39) | **1.85 (1.20-2.85)** |
|  |  |  |  |  |  |  |  |  |  |  |  |  |  |  |
| *Low risk types* |  |  |  |  |  |  |  |  |  |  |  |  |  |  |
| HPV6 | 17 (3.4) | 1 (3.3) | 1.07 (0.13-9.07) | 12 (2.4) | 1 (3.3) | 2.13 (0.19-23.91) | 1.52 (0.30-7.59) | 71 (14.5) | 5 (17.2) | 1.08 (0.35-3.34) | 52 (11.0) | 1 (4.0) | 0.36 (0.05-2.84) | 0.78 (0.28-2.18) |
| HPV11 | 18 (3.4) | 0 (0.0) | (-) | 13 (2.5) | 0 (0.0) | (-) | (-) | 73 (14.8) | 3 (10.7) | 0.62 (0.17-2.21) | 50 (10.7) | 3 (10.7) | 0.59 (0.12-2.80) | 0.62 (0.22-1.76) |

Adjusted Odds Ratio; ^a^adjusted for age, bacterial vaginosis, cervical ectopy, CD4+ count and ART duration in BF; ^b^adjusted for age at first pregnancy, injectable contraception, CD4+ count and ART duration in SA; **^c^**Reference group for any multiple HR-HPV is negative for any HR-HPV *OR* single HR-HPV infection
