# Supplementary material for: Associations of Human Papillomavirus (HPV) genotypes with high-grade cervical neoplasia (CIN2+) in a cohort of women living with HIV in Burkina Faso and South Africa
Source: PLoS One. 2017 Mar 23;12(3):e0174117. doi: 10.1371/journal.pone.0174117 (PMC5363860; doi:10.1371/journal.pone.0174117)
Supplement: S2 Table — (DOCX) [file pone.0174117.s002.docx]

**S2 Table.** Association of HR-HPV type prevalence with ART and CD4+ count at enrolment among 570 women living with HIV in Burkina Faso

|  | **Burkina Faso^a^** | | | | | | |
| --- | --- | --- | --- | --- | --- | --- | --- |
|  |  | **HPV16/18** | | **9vHPV HR^b^** | | **Non vaccine types^c^** | |
|  | **N** | **n (%)** | **aPR (95% CI)** | **n (%)** | **aPR (95% CI)** | **n (%)** | **aPR (95% CI)** |
| On ART | 412 | 63 (15.3) | 1.00 | 123 (32.2) | 1.00 | 69 (30.5) | 1.00 |
| ART-naive | 158 | 22 (13.9) | 0.94 (0.59-1.51) | 51 (37.5) | 1.03 (0.78-1.36) | 25 (29.4) | 0.91 (0.59-1.41) |
|  |  |  |  |  |  |  |  |
| CD4+ count among ALL, cells/mm^3^ | |  |  |  |  |  |  |
| <200 | 66 | 14 (21.2) | 1.44 (0.80-2.60) | 24 (46.2) | **1.52 (1.06-2.18)** | 12 (42.9) | 1.75 (1.00-3.07) |
| 201-350 | 125 | 17 (13.6) | 0.92 (0.53-1.58) | 42 (38.9) | 1.16 (0.84-1.61) | 21 (31.8) | 1.23 (0.76-2.01) |
| 351-500 | 161 | 23 (14.3) | 0.95 (0.57-1.59) | 44 (31.9) | 1.00 (0.72-1.38) | 31 (33.0) | 1.27 (0.82-1.96) |
| >500 | 217 | 31 (14.3) | 1.00 | 63 (33.9) | 1.00 | 30 (24.4) | 1.00 |
|  |  |  |  |  |  |  |  |
| CD4+ count among  ART users, cells/mm^3^ | |  |  |  |  |  |  |
| <200 | 47 | 9 (19.2) | 0.98 (0.47-2.06) | 16 (42.1) | **1.66 (1.07-2.58)** | 11 (50.0) | **2.05 (1.14-3.65)** |
| 201-350 | 80 | 11 (13.8) | 0.75 (0.39-1.43) | 30 (43.5) | 1.42 (0.96-2.10) | 12 (30.8) | 1.32 (0.74-2.35) |
| 351-500 | 119 | 17 (14.3) | 0.75 (0.42-1.35) | 33 (32.4) | 1.16 (0.79-1.72) | 23 (33.3) | 1.23 (0.74-2.05) |
| >500 | 165 | 26 (15.8) | 1.00 | 43 (30.9) | 1.00 | 23 (24.0) | 1.00 |
|  |  |  |  |  |  |  |  |
| CD4+count among  ART-naïve, cells/mm^3^ | |  |  |  |  |  |  |
| <200 | 19 | 5 (26.3) | 2.02 (0.63-6.45) | 8 (57.1) | 1.15 (0.59-2.24) | 1 (16.7) | 0.88 (0.13-5.76) |
| 201-350 | 45 | 6 (13.3) | 1.25 (0.43-3.60) | 12 (30.8) | 0.66 (0.37-1.20) | 9 (33.3) | 1.25 (0.48-3.22) |
| 351-500 | 42 | 6 (14.3) | 1.22 (0.41-3.63) | 11 (30.6) | 0.71 (0.39-1.27) | 8 (32.0) | 1.44 (0.58-3.57) |
| >500 | 52 | 5 (9.6) | 1.00 | 20 (42.6) | 1.00 | 7 (25.9) | 1.00 |

aPR=adjusted Prevalence Ratio; ^a^adjusted for alcohol, marital status, age at first pregnancy and cervicitis in BF; ^b^9vHPV HR includes HPV31/33/45/52/58 in absence of HPV16/18 compared to being negative for all of HPV31/33/45/52/58; ^c^Non vaccine types includes HPV35/39/51/56/59/68 in absence of any nonavalent vaccine type compared to being HR-HPV negative.
