# Supplementary material for: Associations of Human Papillomavirus (HPV) genotypes with high-grade cervical neoplasia (CIN2+) in a cohort of women living with HIV in Burkina Faso and South Africa
Source: PLoS One. 2017 Mar 23;12(3):e0174117. doi: 10.1371/journal.pone.0174117 (PMC5363860; doi:10.1371/journal.pone.0174117)
Supplement: S3 Table — (DOCX) [file pone.0174117.s003.docx]

**S3 Table.** Association of HR-HPV type prevalence with ART and CD4+ count at enrolment among 613 women living with HIV in South Africa

|  | **South Africa^a^** | | | | | | |
| --- | --- | --- | --- | --- | --- | --- | --- |
|  |  | **HPV16/18** | | **9vHPV HR^b^** | | **Non vaccine types^c^** | |
|  | **N** | **n (%)** | **aPR (95%CI)** | **n (%)** | **aPR (95%CI)** | **n (%)** | **aPR (95%CI)** |
| On ART | 404 | 100 (24.8) | 1.00 | 148 (48.7) | 1.00 | 69 (44.2) | 1.00 |
| ART-naive | 209 | 79 (37.8) | **1.42 (1.11-1.83)** | 76 (58.5) | 1.16 (0.96-1.40) | 20 (37.0) | 0.92 (0.62-1.37) |
|  |  |  |  |  |  |  |  |
| CD4+ count among ALL, cells/mm^3^ | |  |  |  |  |  |  |
| <200 | 57 | 17 (29.8) | 1.22 (0.77-1.93) | 23 (57.5) | 1.13 (0.85-1.50) | 10 (58.8) | **1.71 (1.06-2.75)** |
| 201-350 | 148 | 53 (35.8) | 1.47 (1.07-2.01) | 48 (50.5) | 0.90 (0.70-1.17) | 21 (44.7) | 1.27 (0.82-1.95) |
| 351-500 | 183 | 54 (29.5) | 1.18 (0.86-1.63) | 64 (49.6) | 0.97 (0.78-1.20) | 26 (40.0) | 1.12 (0.73-1.70) |
| >500 | 225 | 55 (24.4) | 1.00 | 89 (52.4) | 1.00 | 32 (39.5) | 1.00 |
|  |  |  |  |  |  |  |  |
| CD4+ count among  ART users, cells/mm^3^ | |  |  |  |  |  |  |
| <200 | 51 | 15 (29.4) | 1.57 (0.91-2.73) | 20 (55.6) | 1.05 (0.76-1.46) | 10 (62.5) | **1.82 (1.08-3.05)** |
| 201-350 | 102 | 34 (33.3) | **1.72 (1.09-2.71)** | 34 (50.0) | 0.89 (0.65-1.20) | 16 (47.1) | 1.33 (0.79-2.24) |
| 351-500 | 113 | 26 (23.0) | 1.20 (0.73-1.96) | 35 (40.2) | 0.79 (0.59-1.07) | 23 (44.2) | 1.21 (0.74-1.97) |
| >500 | 138 | 25 (18.1) | 1.00 | 59 (52.2) | 1.00 | 20 (37.0) | 1.00 |
|  |  |  |  |  |  |  |  |
| CD4+count among  ART-naïve, cells/mm^3^ | |  |  |  |  |  |  |
| <200 | 6 | 2 (33.3) | 1.06 (0.34-3.29) | 3 (75.0) | 1.53 (0.83-2.83) | 0 (0.0) | - |
| 201-350 | 46 | 19 (41.3) | 1.26 (0.80-1.98) | 14 (51.9) | 1.03 (0.62-1.70) | 5 (38.5) | 0.86 (0.37-2.00) |
| 351-500 | 70 | 28 (40.0) | 1.26 (0.80-1.98) | 29 (69.1) | **1.45 (1.04-2.01)** | 3 (23.1) | 0.57 (0.19-1.69) |
| >500 | 87 | 30 (34.5) | 1.00 | 30 (52.6) | 1.00 | 12 (44.4) | 1.00 |

aPR=adjusted Prevalence Ratio; ^a^adjusted for age, smoking, injectable contraception, genital warts, condom, vaginal cleansing, *Chlamydia trachomatis, Trichomonas vaginalis* and bacterial vaginosis in SA; ^b^9vHPV HR includes HPV31/33/45/52/58 in absence of HPV16/18 compared to being negative for all of HPV31/33/45/52/58; ^c^Non vaccine types includes HPV35/39/51/56/59/68 in absence of any nonavalent vaccine type compared to being HR-HPV negative.
